# Impact of atrial fibrillation on the development of ischemic stroke among cancer patients classified by CHA_2_DS_2_-VASc score-a nationwide cohort study

**DOI:** 10.18632/oncotarget.24143

**Published:** 2018-01-10

**Authors:** Wei-Syun Hu, Cheng-Li Lin

**Affiliations:** ^1^ School of Medicine, College of Medicine, China Medical University, Taichung 40402, Taiwan; ^2^ Division of Cardiovascular Medicine, Department of Medicine, China Medical University Hospital, Taichung 40447, Taiwan; ^3^ Management Office for Health Data, China Medical University Hospital, Taichung 40447, Taiwan

**Keywords:** atrial fibrillation, cancer, CHA_2_DS_2_-VASc score, ischemic stroke

## Abstract

**Purpose:**

The current study aimed to explore the impact of atrial fibrillation (AF) on risk of ischemic stroke among cancer patients classified by CHA_2_DS_2_-VASc score.

**Methods:**

Study participants were identified from Registry for Catastrophic Illness Patient Database. All cancer patients whether they had comorbid AF or not were divided into 4 groups according to their CHA_2_DS_2_-VASc score-a score of 0–1, 2–3, 4–5 and >5. Competing risk analysis was used to evaluate the subhazard ratios (SHRs) and 95% confidence intervals (CIs) of incident ischemic stroke between cancer patients with and without AF according to their CHA_2_DS_2_-VASc score.

**Results:**

A total of 781473 patients with cancer were identified. Of them, 21134 had comorbid AF whereas the remaining 760339 patients did not. After controlling for the confounding factors and the competing risk of death, among cancer patients, those with AF were associated with the highest risk of ischemic stroke than those without AF while their CHA_2_DS_2_-VASc score was 0∼1 (adjusted SHR [aSHR] = 4.15, 95% CI = 3.29–5.23). Among those with a CHA_2_DS_2_-VASc score of >5, the AF group exhibited a 1.82-fold higher risk of ischemic stroke than the non-AF group (95% CI = 1.34–2.47).

**Conclusions:**

The impact of AF on risk of ischemic stroke was attenuated with advancing CHA_2_DS_2_-VASc score in patients with cancer.

## INTRODUCTION

Currently, cancer and ischemic stroke remain major challenges for the developed countries because of the associated high morbidity and mortality [1–6]. Although the connection between cancer and incident ischemic stroke has been explored, the underlying detailed mechanism link between cancer and ischemic stroke is still not fully understood [1–9]. Several possible factors for the development of incident ischemic stroke among patients with cancer were proposed, mainly through either cancer-related or cancer-unrelated mechanism [1–9].

Atrial fibrillation (AF), a well-recognized risk factor for ischemic stroke, is a common comorbidity in cancer patients [10–12]. In addition, the prognostic impact of AF on the clinical outcomes among patients with cancer has also been investigated [10, 13–14]. Furthermore, current guidelines suggest that CHA_2_DS_2_-VASc score is a useful discrimination tool for stratifying ischemic stroke risk among patients with AF [15–17]. Recently, CHA_2_DS_2_-VASc score has been shown to be helpful for stroke risk stratification even in individuals without AF [18–19].

To our knowledge, currently there is no study specifically addressing the issue of the link between cancer, AF and ischemic stroke. To bridge the gaps of knowledges, the current study aimed to identify the impact of AF on risk of developing ischemic stroke among cancer patients stratified by CHA_2_DS_2_-VASc score. The predictive capacity of CHA_2_DS_2_-VASc scores for stroke risk discrimination among cancer patients whether they have comorbid AF or not was also explored.

## METHODS

### Data source

We undertook a retrospective nationwide cohort study using the Registry of Catastrophic Illness Patient Database (RCIPD) of the Taiwan National Health Insurance (NHI) program. A compulsory NHI program was implemented in Taiwan since 1995 and it covered nearly 99% of all residents (23.74 million beneficiaries) of Taiwan [20]. The details of the RCIPD have been described previously [21–22]. This study complied with the guidelines of the Declaration of Helsinki and was approved by the Institutional Review Board (IRB) of China Medical University and Hospital (CMUH-104-REC2-115). The diagnostic codes were based on the International Classification of Diseases, Ninth Revision, Clinical Modification (ICD-9 codes).

### Sampled participants

All cancer (ICD-9-CM 140–208) patients were identified from RCIPD and the first-time cancer diagnosis served as the index date from 2000 to 2011. The cancer patients were divided into 2 groups, based on with and without a history of atrial fibrillation (AF) (ICD-9-CM code 427.31). Patients with incomplete age or sex information were excluded. The CHA_2_DS_2_-VASc score was calculated for each patient to measure incident ischemic stroke risk [23–26]. Patient’s baseline comorbidities included hyperlipidemia (ICD-9-CM code 272), chronic obstructive pulmonary disease (COPD) (ICD-9-CM code 491, 492, 496), chronic kidney disease (CKD) (ICD-9-CM code 585), hyperthyroidism (ICD-9-CM code 242), sleep disorder (ICD-9-CM codes 307.4 and 780.5), and gout (ICD-9-CM code 274). All subjects were followed until a diagnosis of ischemic stroke (ICD-9-CM codes 433–438) was made, until death, withdrawal from insurance, or the end of 2011, whichever came first.

### Statistical analysis

Demographic characteristics and comorbidity prevalence were compared between the AF and the non-AF groups. The difference of categorical variables between the two groups was examined using the *χ* ^2^ test whereas the difference of continuous variables between the two groups was examined with the *t*-test. Cancer patients with and without AF were divided into 4 groups according to their CHA_2_DS_2_-VASc score-a score of 0–1, 2–3, 4–5 and >5. The incidence of ischemic stroke and death in patients with cancer whether they had comorbid AF or not were estimated according to their CHA_2_DS_2_-VASc score. The cumulative incidence of ischemic stroke stratified by CHA_2_DS_2_-VASc score among cancer patients with and without AF was assessed using the Kaplan-Meier method, and the differences between the curves were evaluated using a log-rank test. To quantify the discriminatory properties of the CHA_2_DS_2_-VASc score in predicting ischemic stroke among cancer patients with and without AF, we plotted the receiver operating characteristic (ROC) curves and calculated the area under the ROC curves. Considering the competing risk of death, the Fine and Gray model was used to extend the standard univariable and multivariable Cox proportional hazards models and the subhazard ratios (SHRs) and 95% confidence intervals (CIs) of incident ischemic stroke between cancer patients with and without AF according to their CHA_2_DS_2_-VASc score were obtained (27). All data processing and statistical analyses were performed with the SAS software version 9.4 (SAS Institute, Inc., Cary, NC, USA). A *p*-value of <0.05 was considered statistically significant.

## RESULTS

A total of 781473 cancer patients were identified from RCIPD. Among them, 21134 patients had comorbid AF whereas 760339 patients did not (Table 1). Most cancer patients with AF were aged 75 years or older (57.1%) whereas most cancer patients without AF were aged 64 years or younger (56.4%). The mean (SD) ages was 75.3 (9.83) and 61.2 (14.9) years in the AF and the non-AF group, respectively. The percentage of women in AF group was significantly higher than the non-AF group (65.2% vs 55.2%, *p <* 0.001). The AF group was associated with a higher prevalence of all comorbidities than the non-AF group. The 3 major cancers in the AF group were colon cancer (18.3%), lung cancer (17.1%) and hepatoma (13.2%) whereas colon cancer (13.8 %), hepatoma (13.4%) and female breast cancer (11.4%) were three most common cancers in the non-AF groups. The mean CHA_2_DS_2_-VASc score of the AF and the non-AF group were 3.86 (SD = 1.72) and 1.89 (SD = 1.56). The mean follow up period for the AF and the non-AF group was 1.79 years (SD = 2.19 years) and 3.00 years (SD = 3.07 years), respectively.

**Table 1:** Baseline characteristics of cancer patients with and without atrial fibrillation

Characteristics	Atrial fibrillation		*p*-value
	No	Yes	
	*N* = 760339	*N* = 21134	
	*n* (%)	*n* (%)	
Age group (year)			<0.001
≤64	429050 (56.4)	2953 (14.0)	
65–74	174396 (22.9)	6112 (28.9)	
≥75	156893 (20.6)	12069 (57.1)	
Age, mean ± SD ^a^ (year)	61.2 ± 14.9	75.3 ± 9.83	<0.001
Sex			<0.001
Women	419477 (55.2)	13778 (65.2)	
Men	340862 (44.8)	7356 (34.8)	
Underlying disease (components of the CHA_2_DS_2_-VASc score)			
HF	34965 (4.60)	8606 (40.7)	<0.001
Diabetes Mellitus	121743 (16.0)	5274 (25.0)	<0.001
CVA or TIA	66492 (8.75)	6432 (30.4)	<0.001
Vascular disease	23472 (3.09)	2484 (11.8)	<0.001
Hypertension	336445 (44.3)	17282 (81.8)	<0.001
Other underlying disease			
Hyperlipidemia	173145 (22.8)	6603 (31.2)	<0.001
COPD	139500 (18.4)	9344 (44.2)	<0.001
Chronic kidney disease	42882 (5.64)	2837 (13.4)	<0.001
Hyperthyroidism	12812 (1.69)	775 (3.67)	<0.001
Sleep disorder	177617 (23.4)	6899 (32.6)	<0.001
Gout	94416 (12.4)	5134 (24.3)	<0.001
Type of Cancer (ICD-9-CM)			
Hematologic malignancy (200–208)	34301 (4.51)	1056 (5.00)	0.001
Head and neck (140–149, 161)	80000 (10.5)	1000 (4.73)	<0.001
Esophagus (150)	16853 (2.22)	455 (2.15)	0.54
Stomach (151)	37434 (4.92)	1446 (6.84)	<0.001
Colon (153–154)	104751 (13.8)	3858 (18.3)	<0.001
Hepatoma (155)	101416 (13.4)	2799 (13.2)	0.69
Cholangiocarcinoma (156)	8958 (1.18)	329 (1.56)	<0.001
Pancreas (157)	12803 (1.68)	436 (2.06)	<0.001
Lung (162)	84792 (11.2)	3622 (17.1)	<0.001
Skin (173)	11688 (1.54)	469 (2.22)	<0.001
Female breast (174)	86451 (11.4)	775 (3.67)	<0.001
Uterus (180–184)	49496 (6.51)	595 (2.82)	<0.001
Prostate (185)	33304 (4.38)	1762 (8.34)	<0.001
Bladder & Kidney (188, 189)	42664 (5.61)	1485 (7.03)	<0.001
Brain (191)	8429 (1.11)	170 (0.80)	<0.001
Thyroid (193)	20158 (2.65)	233 (1.10)	<0.001
Mean CHA_2_DS_2_-VASc score (SD)^a^	1.89 (1.56)	3.86 (1.72)	<0.001
Mean follow-up, y (SD)^a^	3.00 (3.07)	1.79 (2.19)	<0.001

Chi-square test.

^a^*t*-test.

SD denotes standard difference.

HF denotes heart failure.

CVA denotes cerebrovascular accident.

TIA denotes transient ischemic attack.

COPD denotes chronic obstructive pulmonary disease.

**Figure 1:**
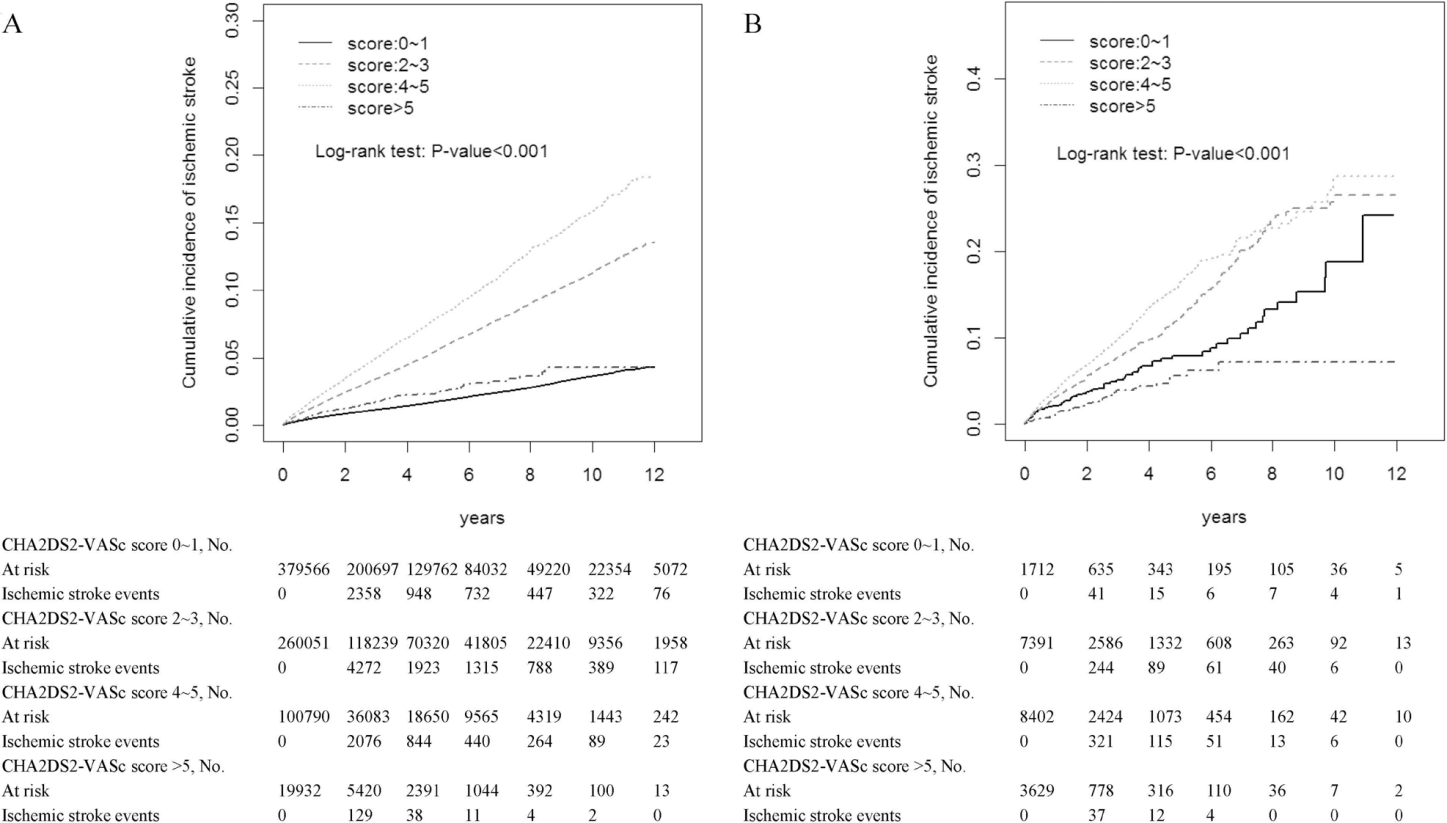
Cumulative incidence curves of incident ischemic stroke according to CHA_2_DS_2_-VASc score in cancer patients without atrial fibrillation **(A)** and with atrial fibrillation **(B)**.

**Figure 2:**
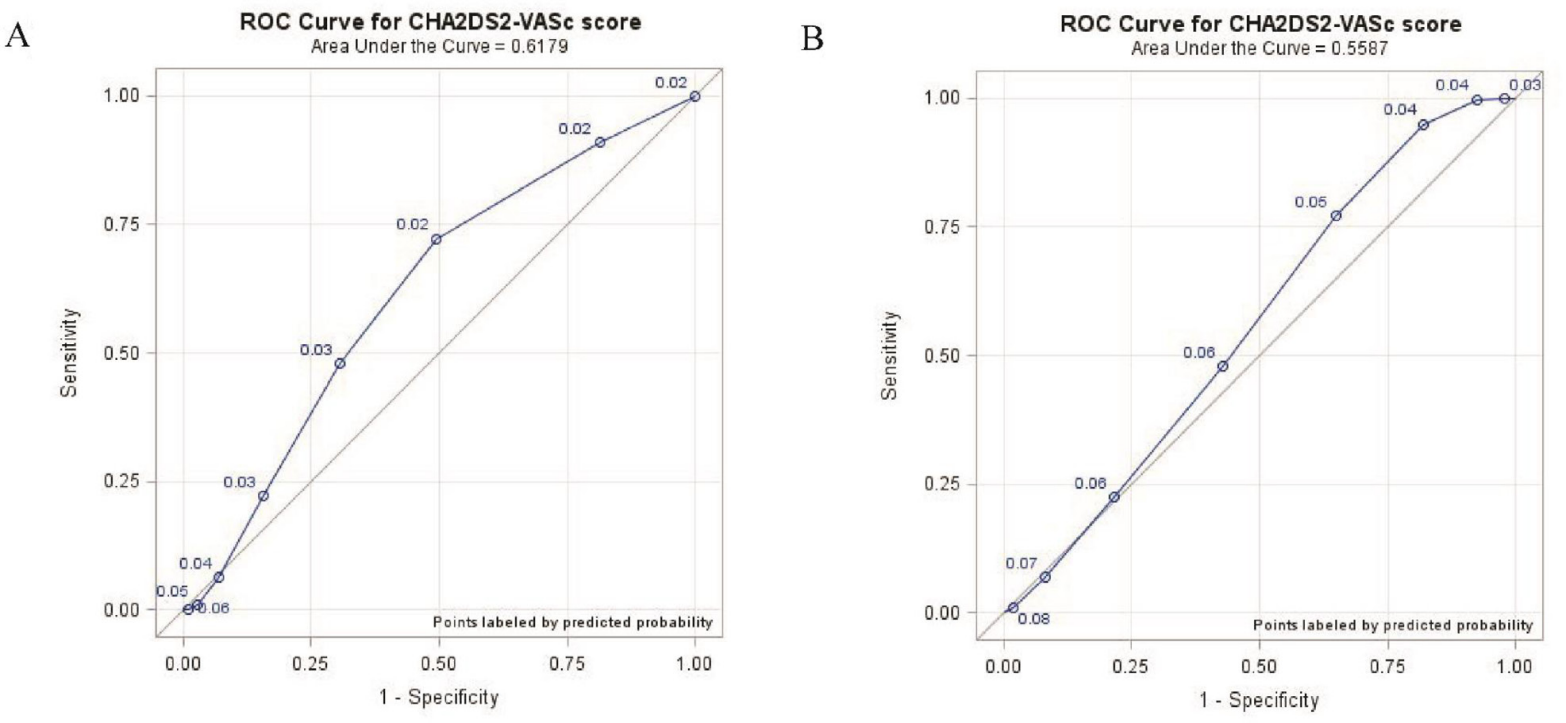
Receiver operating characteristic (ROC) curve for CHA_2_DS_2_-VASc score in predicting incident ischemic stroke in cancer patients without atrial fibrillation **(A)** and with atrial fibrillation **(B)**.

The incidence of ischemic stroke was highest in cancer patients whether they had comorbid AF or not while their CHA_2_DS_2_-VASc score was 4∼5 (6.02% vs 3.71%) (Table 2). Kaplan-Meier analyses illustrated the cumulative incidence curves of incident ischemic stroke according to CHA_2_DS_2_-VASc score in cancer patients without atrial fibrillation (Figure 1A) and with atrial fibrillation (Figure 1B). Among cancer patients without and with comorbid AF, the area under the ROC curves of the CHA_2_DS_2_-VASs score as a predictor of ischemic stroke was 0.62 (95% CI = 0.61–0.62) (Figure 2A) and 0.56 (95% CI = 0.54–0.57) (Figure 2B), respectively.

**Table 2:** The incidence of ischemic stroke according to CHA_2_DS_2_-VASc score among cancer patients with and without AF

	Atrial fibrillation					
	No			Yes		
CHA_2_DS_2_-VASc score	N	Event	Incidence (%)	N	Event	Incidence (%)
0∼1	379566	4887	1.29	1712	74	4.32
2∼3	260051	8809	3.39	7391	440	5.95
4∼5	100790	3736	3.71	8402	506	6.02
>5	19932	184	0.92	3629	54	1.49

The incidence of mortality was highest in cancer patients whether or not AF was present while their CHA_2_DS_2_-VASc score was greater than 5 (49.5% vs 47.0%) (Table 3). Estimation of incident ischemic stroke risk in the study cohorts while considering the competing risk of death was shown in Table 4. After controlling for the confounding factors and the competing risk of death, among cancer patients, those with comorbid AF were associated with the highest risk of ischemic stroke than those without AF while their CHA_2_DS_2_-VASc score was 0∼1 (adjusted SHR [aSHR] = 4.15, 95% CI = 3.29–5.23). Additionally, among cancer patients with a CHA_2_DS_2_-VASc score of >5, the AF group exhibited a 1.82-fold higher risk of ischemic stroke than the non-AF group (95% CI = 1.34–2.47).

**Table 3:** The incidence of mortality according to CHA_2_DS_2_-VASc score among cancer patients with and without AF

	Atrial fibrillation					
	No			Yes		
CHA_2_DS_2_-VASc score	*N*	Event	Incidence (%)	*N*	Event	Incidence (%)
0∼1	379566	112614	29.7	1712	685	40.0
2∼3	260051	96386	37.1	7391	3232	43.7
4∼5	100790	43240	42.9	8402	3903	46.5
>5	19932	9372	47.0	3629	1795	49.5

**Table 4:** Incidence and subhazard ratios of ischemic stroke between cancer patients with and without atrial fibrillation according to CHA_2_DS_2_-VASc score with the use of the competing-risk regression model

	Atrial fibrillation							
	No				Yes			
CHA_2_DS_2_-VASc score	Event	Rate^#^	Crude SHR (95% CI)	Adjusted SHR^†^ (95% CI)	Event	Rate^#^	Crude SHR (95% CI)	Adjusted SHR^†^ (95% CI)
0∼1	4887	37.6	1 (Reference)	1 (Reference)	74	188.8	4.67 (3.71, 5.87)^***^	4.15 (3.29, 5.23)^***^
2∼3	8809	120.2	1 (Reference)	1 (Reference)	440	290.1	1.98 (1.80, 2.18)^***^	1.95 (1.77, 2.14)^***^
4∼5	3736	174.7	1 (Reference)	1 (Reference)	506	362.5	1.84 (1.67, 2.01)^***^	1.86 (1.69, 2.04)^***^
>5	184	57.9	1 (Reference)	1 (Reference)	54	114.3	1.78 (1.32, 2.41)^***^	1.82 (1.34, 2.47)^***^

Rate^#^, per 1,000 person-year; Crude SHR, crude subhazard ratio.

^†^Adjusted for hyperlipidemia, chronic obstructive pulmonary disease, chronic kidney disease, hyperthyroidism, sleep disorder and gout; further controlling for death to estimate adjusted subhazard ratio.

^***^p < 0.001.

## DISCUSSION

We reported for the first time regarding the impact of AF on incident ischemic stroke among cancer patients stratified by CHA_2_DS_2_-VASc score. Additionally, the use of CHA_2_DS_2_-VASc score in ischemic stroke risk discrimination among cancer patients with and without comorbid AF was also addressed.

Using CHA_2_DS_2_-VASc score for stratifying patients into four groups (0–1, 2–3, 4–5, and >5), the incidence of mortality increased in parallel with increasing score in cancer patients whether AF was present or not. Nevertheless, the linear shape phenomenon was not observed in the incident ischemic stroke among cancer patients with and without AF. Possible factor for the explanation is that death is a major competing risk for ischemic stroke in cancer patients, leading to the lowest incidence of ischemic stroke in those with the highest score [1–6].

The biologic mechanism link between cancer and stroke is complicated and currently not comprehensively elucidated [1–9]. Either cancer-related mechanism, such as hypercoagulable status, tumor compression, paradoxical embolism, nonbacterial thrombotic endocarditis and anti-cancer therapy associated effect or cancer-unrelated mechanism, such as overlapping atherosclerotic risk factors were proposed [1–9].

In the current study, we found that cancer patients with AF were older and had more underlying medical comorbidities. After controlling for the potential confounding factors with the competing risk regression model approach, the risk of developing ischemic stroke for cancer patients with AF relative to those without AF was in an inverse relationship with advancing CHA_2_DS_2_-VASc score. Possible explanation for our results is that a higher CHA_2_DS_2_-VASc score is associated with a significantly higher rate of ischemic stroke, which in turn might lead to the attenuated role of AF in those with a higher score.

Several advantages of the current study deserve to be highlighted. First, the study was analyzed from a large insurance administrative database which has been validated [28–30]. Second, this study is a large scale investigation with large patient population and reasonable follow up duration with the attempt to explore this clinically significant issue. Finally, statistical procedure with competing risk analysis approach was used in order to minimize the study bias [27].

### Limitations

First, the absence of relevant case information from the insurance claims data is potentially a major limitation and should be outlined. However, several validation studies have been conducted and the reliability of the nationwide dataset has been confirmed [28–30]. Second, detailed personal health-associated behaviors were uncertain in this database. Finally, potential unmeasured confounding variables involved in the analyses are possible. Despite the limitations presented here, this study was extracted from a large nationwide dataset with the appropriate methodology approach to provide new insights into this context.

## CONCLUSIONS

The impact of AF on risk of ischemic stroke was attenuated with the advancement of CHA_2_DS_2_-VASc score in cancer patients.

## References

[1] Navi BB, Reiner AS, Kamel H, Iadecola C, Elkind MS, Panageas KS, DeAngelis LM (2015). Association between incident cancer and subsequent stroke. Ann Neurol.

[2] Selvik HA, Thomassen L, Bjerkreim AT, Næss H (2015). Cancer-Associated Stroke: The Bergen NORSTROKE Study. Cerebrovasc Dis Extra.

[3] Dearborn JL, Urrutia VC, Zeiler SR (2014). Stroke and Cancer-A Complicated Relationship. J Neurol Transl Neurosci.

[4] Schwarzbach CJ, Schaefer A, Ebert A, Held V, Bolognese M, Kablau M, Hennerici MG, Fatar M (2012). Stroke and cancer: the importance of cancer-associated hypercoagulation as a possible stroke etiology. Stroke.

[5] Bang OY, Seok JM, Kim SG, Hong JM, Kim HY, Lee J, Chung PW, Park KY, Kim GM, Chung CS, Lee KH (2011). Ischemic stroke and cancer: stroke severely impacts cancer patients, while cancer increases the number of strokes. J Clin Neurol.

[6] Kim SG, Hong JM, Kim HY, Lee J, Chung PW, Park KY, Kim GM, Lee KH, Chung CS, Bang OY (2010). Ischemic stroke in cancer patients with and without conventional mechanisms: a multicenter study in Korea. Stroke.

[7] Qureshi AI, Malik AA, Saeed O, Adil MM, Rodriguez GJ, Suri MF (2015). Incident cancer in a cohort of 3,247 cancer diagnosis free ischemic stroke patients. Cerebrovasc Dis.

[8] Kim SJ, Park JH, Lee MJ, Park YG, Ahn MJ, Bang OY (2012). Clues to occult cancer in patients with ischemic stroke. PLoS One.

[9] Chen PC, Muo CH, Lee YT, Yu YH, Sung FC (2011). Lung cancer and incidence of stroke: a population-based cohort study. Stroke.

[10] Farmakis D, Parissis J, Filippatos G (2014). Insights into onco-cardiology: atrial fibrillation in cancer. J Am Coll Cardiol.

[11] Conen D, Wong JA, Sandhu RK, Cook NR, Lee IM, Buring JE, Albert CM (2016). Risk of Malignant Cancer Among Women With New-Onset Atrial Fibrillation. JAMA Cardiol.

[12] Ostenfeld EB, Erichsen R, Pedersen L, Farkas DK, Weiss NS, Sørensen HT (2014). Atrial fibrillation as a marker of occult cancer. PLoS One.

[13] Walsh SR, Gladwish KM, Ward NJ, Justin TA, Keeling NJ (2004). Atrial fibrillation and survival in colorectal cancer. World J Surg Oncol.

[14] Hu YF, Liu CJ, Chang PM, Tsao HM, Lin YJ, Chang SL, Lo LW, Tuan TC, Li CH, Chao TF, Chung FP, Liao JN, Chen TJ (2013). Incident thromboembolism and heart failure associated with new-onset atrial fibrillation in cancer patients. Int J Cardiol.

[15] January CT, Wann LS, Alpert JS, Calkins H, Cigarroa JE, Cleveland JC, Conti JB, Ellinor PT, Ezekowitz MD, Field ME, Murray KT, Sacco RL, Stevenson WG (2014). American College of Cardiology/American Heart Association Task Force on Practice Guidelines. 2014 AHA/ACC/HRS guideline for the management of patients with atrial fibrillation: a report of the American College of Cardiology/American Heart Association Task Force on Practice Guidelines and the Heart Rhythm Society. J Am Coll Cardiol.

[16] Camm AJ, Lip GY, De Caterina R, Savelieva I, Atar D, Hohnloser SH, Hindricks G, Kirchhof P, ESC Committee for Practice Guidelines (CPG) (2012). 2012 focused update of the ESC Guidelines for the management of atrial fibrillation: an update of the 2010 ESC Guidelines for the management of atrial fibrillation. Developed with the special contribution of the European Heart Rhythm Association. Eur Heart J.

[17] Fuster V, Rydén LE, Cannom DS, Crijns HJ, Curtis AB, Ellenbogen KA, Halperin JL, Kay GN, Le Huezey JY, Lowe JE, Olsson SB, Prystowsky EN, Tamargo JL (2011). 2011 ACCF/AHA/HRS focused updates incorporated into the ACC/AHA/ESC 2006 Guidelines for the management of patients with atrial fibrillation: a report of the American College of Cardiology Foundation/American Heart Association Task Force on Practice Guidelines developed in partnership with the European Society of Cardiology and in collaboration with the European Heart Rhythm Association and the Heart Rhythm Society. Am Coll Cardiol.

[18] Podolecki T, Lenarczyk R, Kowalczyk J, Swierad M, Swiatkowski A, Jedrzejczyk E, Chodor P, Zielinska T, Kalarus Z (2015). Stroke and death prediction with CHA_2_DS_2_-vasc score after myocardial infarction in patients without atrial fibrillation. J Cardiovasc Med (Hagerstown).

[19] Ntaios G, Lip GY, Makaritsis K, Papavasileiou V, Vemmou A, Koroboki E, Savvari P, Manios E, Milionis H, Vemmos K (2013). CHA_2_DS_2_, CHA_2_S_2_DS_2_-VASc, and long-term stroke outcome in patients without atrial fibrillation. Neurology.

[20] National Health Research Institutes National Health Insurance Research Database. http://nhird.nhri.org.tw/en/index.html.

[21] Peng YC, Lin CL, Hsu WY, Chang CS, Yeh HZ, Tung CF, Wu YL, Sung FC, Kao CH (2015). Statins are associated with a reduced risk of cholangiocarcinoma: a population-based case-control study. Br J Clin Pharmacol.

[22] Huang KW, Kuan YC, Luo JC, Lin CL, Liang JA, Kao CH (2016). Impact of long-term gastric acid suppression on spontaneous bacterial peritonitis in patients with advanced decompensated liver cirrhosis. Eur J Intern Med.

[23] Hu WS, Lin CL (2017). CHA_2_DS_2_-VASc score for ischaemic stroke risk stratification in patients with chronic obstructive pulmonary disease with and without atrial fibrillation: a nationwide cohort study. Europace.

[24] Hu WS, Lin CL (2017). Postoperative ischemic stroke and death prediction with CHA_2_DS_2_-VASc score in patients having coronary artery bypass grafting surgery: A nationwide cohort study. Int J Cardiol.

[25] Chao TF, Liu CJ, Liao JN, Wang KL, Lin YJ, Chang SL, Lo LW, Hu YF, Tuan TC, Chung FP, Chen TJ, Lip GY, Chen SA (2016). Use of Oral Anticoagulants for Stroke Prevention in Patients With Atrial Fibrillation Who Have a History of Intracranial Hemorrhage. Circulation.

[26] Chao TF, Liu CJ, Wang KL, Lin YJ, Chang SL, Lo LW, Hu YF, Tuan TC, Chen TJ, Lip GY, Chen SA (2014). Using the CHA_2_DS_2_-VASc score for refining stroke risk stratification in ‘low-risk’ Asian patients with atrial fibrillation. J Am Coll Cardiol.

[27] Fine JP, Gray RJ (1999). A Proportional Hazards Model for the Subdistribution of a Competing Risk. Journal of the American Statistical Association.

[28] Cheng CL, Kao YH, Lin SJ, Lee CH, Lai ML (2011). Validation of the national health insurance research database with ischemic stroke cases in Taiwan. Pharmacoepidemiol Drug Saf.

[29] Cheng CL, Lee CH, Chen PS, Li YH, Lin SJ, Yang YH (2014). Validation of acute myocardial infarction cases in the national health insurance research database in Taiwan. J Epidemiol.

[30] Cheng CL, Chien HC, Lee CH, Lin SJ, Yang YH (2015). Validity of in-hospital mortality data among patients with acute myocardial infarction or stroke in National Health Insurance Research Database in Taiwan. Int J Cardiol.

